# Management of Patients with Early Myelofibrosis: A Discussion of Best Practices

**DOI:** 10.1007/s11899-024-00729-8

**Published:** 2024-03-05

**Authors:** Prithviraj Bose

**Affiliations:** Department of Leukemia, The University of Texas MD Anderson Cancer Center, Houston, TX 77030 USA

**Keywords:** Myelofibrosis, Ruxolitinib, Janus kinase, Myeloproliferative neoplasm

## Abstract

**Purpose of Review:**

Summarize best practices for management of patients with early myelofibrosis (MF).

**Recent Findings:**

Myelofibrosis is a progressive myeloproliferative neoplasm (MPN) that generally produces burdensome symptoms and ultimately leads to worse overall survival than that observed in healthy controls or patients with other MPNs. Several Janus kinase inhibitors and various interferon formulations are now available for treatment of MF, with ruxolitinib notable for extending overall survival in addition to improving MF signs and symptoms.

**Summary:**

The chronic nature of the disease can lead some patients to avoid immediate treatment in favor of a watch-and-wait approach. This review summarizes the patient management approach taken in my practice, providing guidance and a discussion of best practices with an emphasis on the importance and clinical benefits of active treatment in early MF. In particular, a case is made to consider treatment with ruxolitinib for patients with intermediate-1 risk disease and to minimize delay between diagnosis and treatment initiation for patients with intermediate or high-risk disease.

## Introduction

Myelofibrosis (MF) is a progressive myeloproliferative neoplasm (MPN) characterized by bone marrow reticulin and/or collagen fibrosis with megakaryocytic proliferation and atypia [1]. Somatic driver mutations in *JAK2*, *CALR*, or *MPL* occur in most patients, perpetuating cytokine signaling via the Janus kinase/signal transducer and activator of transcription (JAK-STAT) pathway and activating cell proliferation, survival, and several inflammatory pathways [2–5]. The resulting clinical manifestations include anemia, splenomegaly, and hepatomegaly [6], with the disease ultimately leading to reduced overall survival (OS) compared with healthy controls and patients with other MPNs (e.g., essential thrombocythemia (ET) and polycythemia vera (PV)) [7, 8]. In addition, patients experience various burdensome symptoms—including fatigue, abdominal discomfort, night sweats, bone pain, and pruritus—that negatively impact quality of life [9]. The estimated prevalence of MF ranges from 2 to 4 per 100,000 worldwide [10] and from 4 to 6 per 100,000 in the United States [11].

For the purposes of this review, “early MF” is a clinical concept that can be defined in 2 general ways, by either risk of death or disease duration. It should be noted that the World Health Organization classification for MPNs also includes a distinct category for prefibrotic primary MF (a pathologic diagnosis distinct from overt MF and ET) [1]; however, the guidance here is focused on overt MF. Patients with overt MF are risk-stratified, often into low, intermediate-1 (int-1), int-2, or high-risk disease [12]. Currently approved treatments for MF include the JAK inhibitors ruxolitinib (approved for intermediate or high-risk MF) [13], fedratinib (int-2 or high-risk MF) [14], pacritinib (intermediate or high-risk MF with platelet count < 50 × 10^9^/L) [15], and momelotinib (intermediate or high-risk MF and anemia) [16]. Multiple studies have demonstrated that treating MF earlier in the disease course, while the patient is still at int-1 risk status or as soon as possible after diagnosis for intermediate and high risk, may be beneficial to patients, resulting in higher relative efficacy and lower toxicity compared with treating later in the disease course [17•, 18•, 19•, 20••, 21–23, 24••, 25–27].

This review provides guidance and a discussion of best practices for early management of patients with overt MF, including treatment early in the disease course and the importance of initiating treatment promptly rather than watch-and-wait approaches.

## Sample Patient

A 64-year-old female patient presented with symptoms of mild fatigue, moderate night sweats, abdominal pain, and early satiety/fullness of 4 months’ duration. She also reported unexplained weight loss of 12 lbs. The patient had no known comorbidities and had a palpable spleen 8 cm below the left costal margin. Genetic testing showed a *JAK2*V617F mutation. Karyotype was 46XX, and bone marrow biopsy showed megakaryocyte proliferation and atypia with evidence of reticulin fibrosis. Blood work revealed leukoerythroblastosis on the peripheral blood smear, with a red blood cell count of 3.4 × 10^12^/L, white blood cell count of 23.0 × 10^9^/L, and platelet count of 450 × 10^9^/L; hemoglobin was 13.2 g/dL, hematocrit was 36%, mean corpuscular volume was 94 fL, and peripheral blasts were 1%. Diagnosis was determined to be primary MF, with Dynamic International Prognostic Scoring System (DIPSS) risk of int-1, and Mutation-Enhanced International Prognostic Scoring System (MIPSS)70 classification of intermediate risk.

## Patient Workup

**Fig. 1:**
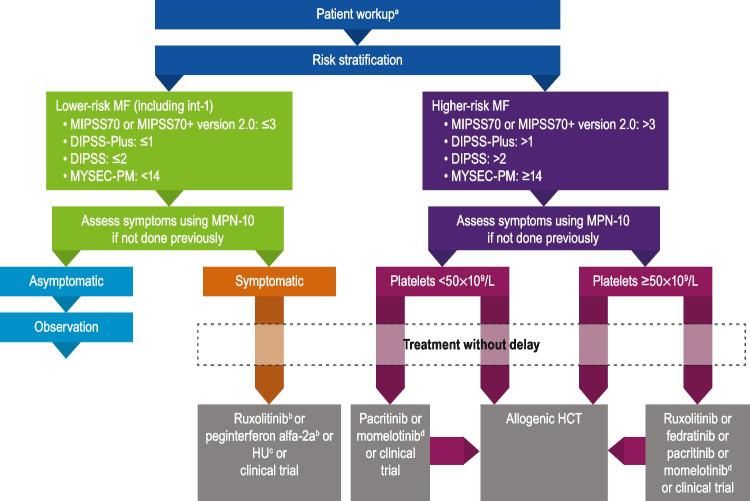
Approach to patient workup, risk stratification, and early treatment in patients with myelofibrosis. DIPSS, Dynamic International Prognostic Scoring System; HCT, hematopoietic cell transplantation; HU, hydroxyurea; int-1, intermediate 1; MF, myelofibrosis; MIPSS, Mutation-Enhanced International Prognostic Scoring System; MPN-10, Myeloproliferative Neoplasm Symptom Assessment Form 10-question version; MYSEC-PM, Myelofibrosis Secondary to Polycythemia Vera and Essential Thrombocythemia-Prognostic Model. ^a^Includes disease history, physician examination, spleen size assessment, evaluation of thrombotic/hemorrhagic events and cardiovascular risk factors, complete blood count, mutational testing, bone marrow biopsy. ^b^Useful in certain circumstances. ^c^If cytoreduction would be symptomatically beneficial. ^d^In patients with anemia and PLT counts ≥ 25 × 10^9^/L

My practice starts with a standard workup that includes patient history and physical examination, spleen size assessment by palpation, evaluation of thrombotic and hemorrhagic events and cardiovascular risk factors, complete blood count, and bone marrow biopsy, including molecular testing for *JAK2/CALR/MPL* (Fig. 1) [12]. We also perform molecular testing for prognostically adverse molecular markers (e.g., mutated *ASXL1*, *EZH2*, *SRSF2*, *U2AF1* [*Q157*], *or IDH1/2*, and unfavorable karyotypes [trisomy 8, − 7/7q − , i(17q), − 5/5q − , 12p − , inv(3), or 11q23 rearrangement]). Additionally, patients in my practice are asked to fill out an online questionnaire in their electronic health record to systematically capture presence and severity of MF symptoms via self-administration of the MPN Symptom Assessment Form (MPN-SAF) [28] before their visit, for efficiency and convenience. Complete findings from the initial workup are used to establish the patients’ risk status. Although by some estimates, ~ 40% of patients receive inaccurate risk categorization and an additional 30% receive no risk categorization at diagnosis [29], establishing accurate risk stratification of MF is an important first step to ensure patients receive proper care. My preferred prognostic risk models are the MIPSS70 + version 2.0 [30] for primary MF and the Myelofibrosis Secondary to PV and ET-Prognostic Model (MYSEC-PM) [31] for prognostication of patients with MF secondary to PV or ET (Table 1).

**Table 1:** Prognostic risk models in myelofibrosis

Parameters (points)	MIPSS70 + v2.0 [67]	MYSEC-PM [31]
Age, y	–	Years × 0.15
Circulating blasts, %	≥ 2 (1)	≥ 3 (2)
Platelets, × 10^9^/L	–	< 150 (1)
Hemoglobin, g/dL	Moderate anemia (1) Severe anemia (2)^a^	< 11 (2)
Constitutional symptoms	Yes (2)	Yes (1)
Cytogenetics	Unfavorable (3) Very high risk (4)^b^	–
Driver mutations	Non-*CALR* type 1 (2)	Non-*CALR* (2)
Other mutations	HMR present (2)^c^ ≥ 2 HMR mutations (3)	–
Risk status (total score)	Very low (0) Low (1–2) Int (3–4) High (5–8) Very high (≥ 9)	Low (< 11) Int-1 (11–13) Int-2 (14–15) High (≥ 16)

*HMR* high molecular risk, *MIPSS70* Mutation-enhanced International Prognostic Scoring System, *MYSEC-PM* Myelofibrosis Secondary to Polycythemia Vera and Essential Thrombocythemia Prognostic Model

^a^Severe: hemoglobin < 8 g/dL in women and < 9 g/dL in men; moderate: hemoglobin 8–9.9 g/dL in women and 9–10.9 g/dL in men

^b^Unfavorable karyotype: any abnormal karyotype other than normal karyotype or sole abnormalities of 20q-, 13q-, + 9, chromosome 1 translocation/duplication, or -Y or sex chromosome abnormality other than –Y; very-high-risk karyotype: single/multiple abnormalities of –7, i(17q), inv(3)/3q21, 12p–/12p11.2, 11q–/11q23, or other autosomal trisomies not including + 8/ + 9 (e.g., + 21, + 19)

^c^Presence of a mutation in any of the following genes: *ASXL1*, *EZH2*, *SRSF2*, *U2AF1 Q157*, or *IDH1/2*

## Molecular Diagnostics and Management Before Disease Progression to MF

Data exist suggesting that active treatment may inhibit progression of PV/ET to MF in the first place. A recent analysis of patients with PV and ET reported that 18% of those treated with ruxolitinib had a sustained molecular response (*JAK2*V617F allele burden < 2%) during long-term treatment, with an associated lower risk of progression to secondary MF [32]. Similarly, in the MAJIC-PV study, patients with PV who were treated with ruxolitinib had more frequent and larger reductions in *JAK2*V617F allele burden than those treated with best available therapy [33]. Molecular response in MAJIC-PV (≥ 50% reduction in *JAK2*V617F allele burden) was associated with better progression-free survival (which included transformation to MF, myelodysplastic syndrome, acute myeloid leukemia, or death from any cause) and event-free survival for patients receiving ruxolitinib, but not those receiving best available therapy. Median time to molecular response in MAJIC-PV was 36 months, with early molecular response at 12 months associated with improved outcomes [33]. Taken together, these data highlight a possible role for early treatment with ruxolitinib, particularly in the more indolent MPN phases to help prevent or delay disease progression to MF.

## Patients with Lower-Risk Disease

Many patients with lower-risk MF have burdensome signs and symptoms and would benefit from active treatment. Real-world data collected at enrollment in the observational MOST study, in which 41% of patients had DIPSS low-risk MF and 59% had int-1 MF, showed that 35% of patients had splenomegaly at enrollment, including 41% with low-risk MF and 31% with int-1–risk MF [34]. The percentages of patients with physician-reported signs or symptoms were similar for both risk groups, with the most common findings being fatigue (30%), lactate dehydrogenase above upper limit of normal (25%), palpable spleen (19%), and leukocytosis (15%). Additionally, disease progression was observed in 24% of the population, including 22% and 25% of those with low-risk and int-1–risk disease, respectively [35]. Of note, 33% of patients in the low-risk group were receiving ruxolitinib monotherapy at enrollment, as well as 46% of those with int-1 MF. Future analyses from MOST should provide further insights into the outcomes and management strategies used in patients with lower-risk MF in US academic and community-based practices.

In my practice, we prefer active treatment with ruxolitinib for symptomatic patients with lower-risk MF, consistent with recommendations from the National Comprehensive Cancer Network (NCCN; Fig. 1) [12]. Formal guidelines to choose between ruxolitinib and interferon formulations are not feasible given the available evidence; the treatment regimen is generally based on clinical decision-making on a case-by-case basis in conjunction with the patient and dependent on their individual situation and characteristics. In my practice, we generally favor using ruxolitinib to address MF-related symptoms and tend to avoid interferon formulations in patients who have depression or autoimmune conditions. Supporting clinical trial data for ruxolitinib use in patients with int-1–risk MF are shown in Table 2. In the ROBUST and JUMP studies, patients with int-1–risk disease experienced improvements in spleen length and symptom severity after treatment with ruxolitinib [17•, 18•, 19•], consistent with data on patients with int-2– or high-risk disease from the phase 3 COMFORT studies [22, 25]. Additional support for early use of ruxolitinib comes from multiple studies by Palandri et al., in which earlier initiation of ruxolitinib (i.e., in int-1 disease) was associated with better efficacy outcomes, including spleen response and symptom resolution, compared with later initiation (i.e., in higher-risk disease) [20••, 23, 27].

**Table 2:** Efficacy benefits of early treatment with ruxolitinib

Study	Key findings
Benefits of ruxolitinib use in int-1 patients	
ROBUST	• At week 48: 50% of int-1 patients achieved a ≥ 50% reduction from BL in spleen length; 21% achieved a ≥ 50% improvement in symptom severity [19•]
JUMP	• At week 48: 61% of int-1 patients achieved a ≥ 50% reduction from BL in spleen length • Clinically meaningful improvements in symptom severity from BL as early as 4 weeks and maintained through Week 48 [17•, 18•]
Palandri et al. (2018) (*Hematol Oncol*)	• At 6 months: 55% of int-1 patients achieved a ≥ 50% reduction from BL in spleen length; 80% achieved a ≥ 50% reduction in MF-SAF total symptoms score [20••]
Palandri et al. (2018) (*Leuk Res*)	• Higher probability of spleen response vs higher-risk MF in a multivariate analysis (*P* = 0.01) [27]
Benefits of minimizing delay between diagnosis and ruxolitinib initiation	
COMFORT-I	• Decreased spleen volumes and symptom scores vs PBO (i.e., delayed active treatment) in primary analysis (*P* < 0.001 for both) [25] • Longer OS in 5-y analysis (*P* = 0.025 ruxolitinib vs PBO) despite crossover from PBO to ruxolitinib [46]
COMFORT-II	• Decreased spleen volumes (*P* < 0.001) and symptom scores vs BAT (i.e., delayed targeted treatment with JAK inhibitor) in primary analysis [22]
Pooled COMFORT analyses	• Longer 5-y OS vs PBO or BAT (*P* = 0.007) despite crossover from control to ruxolitinib [26] • Better spleen response (*P* = 0.015 at Week 48), fewer anemia and thrombocytopenia events, and longer OS (*P* = 0.043) if ruxolitinib initiated ≤ 12 vs > 12 months from diagnosis [24••]
COMFORT-I long-term follow-up analysis	• Allele burden reductions were greater in patients with shorter vs longer duration between diagnosis and treatment [21]
Palandri et al. (2017)	• > 2-year delay in starting ruxolitinib associated with lower probability of spleen response (*P* = 0.048) and trend toward delayed symptom response (*P* = 0.056) in a multivariate analysis [23]

*BAT* best available therapy, *BL* baseline, *int-1* intermediate-1 myelofibrosis, *MF* myelofibrosis, *OS* overall survival, *PBO* placebo

Monotherapy with recombinant or pegylated interferon alfa-2a can also be used for MF, mainly in patients without splenomegaly or symptoms. Recombinant pegylated interferon was reported to be associated with reductions in bone marrow fibrosis and treatment response in patients without high-risk driver mutations, suggesting that this intervention provided clinical benefit in selected patients with early-stage disease [36, 37]. Recently presented results in patients with prefibrotic or low- or int-1–risk overt MF suggest that ropeginterferon alfa-2a is active in this population as well [38].

Combination treatment with ruxolitinib and pegylated interferon alfa-2a is also an option for certain patients with MF. The phase 1/2 Ruxopeg study showed that this combination was well tolerated and provided reductions in spleen length and allele burden [39]. The phase 2 COMBI study also reported improvements in multiple efficacy parameters and acceptable toxicity in some patients with low- or intermediate-risk MF treated with ruxolitinib and low-dose pegylated interferon alfa-2a, although many patients in the study were intolerant of or refractory to pegylated interferon alfa-2a monotherapy [40]. However, the place of ruxolitinib-interferon combinations in therapy of MF remains unclear, and currently, the best setting for such combination treatment is in the context of a clinical trial.

Finally, an important consideration when debating whether to actively treat lower-risk patients is that initiating ruxolitinib early does not mean efficacy will wane over time, and it does not preclude long-term treatment (i.e., patients do not “use up” ruxolitinib treatment by starting early). For example, many patients in JUMP received ruxolitinib for > 1 year, including 30% who were treated for > 2 years and 13% who were treated for > 3 years [17•]. In a retrospective patient chart review study that evaluated the characteristics of patients with MF who received ruxolitinib for ≥ 3 years, most (84%) received ruxolitinib as the first-line therapy, with median time from presentation to ruxolitinib initiation of 4 months and a starting dose ≥ 10 mg twice daily (bid) in 97% of patients [41]. Overall, 40% of patients treated with ruxolitinib for ≥ 3 years were alive 10 years after initiation, with older age and neutrophil percentage the only predictive factors affecting OS in a multivariate analysis. Taken together, these findings suggest that long-term treatment with ruxolitinib is beneficial when feasible. Ruxolitinib can also be reintroduced after treatment discontinuation to provide additional clinical benefit, including improvements in splenomegaly and MF-related symptoms [18•, 42, 43]. Furthermore, JAK inhibitors in general can be used sequentially, such that lack of response to one agent does not preclude positive outcomes with another [44, 45].

## Patients with Intermediate-2 or High-Risk MF at Diagnosis

In my practice, we evaluate all higher-risk patients with MF for allogeneic hematopoietic cell transplantation (HCT), consistent with NCCN guidelines (Fig. 1) [12]. Selection for HCT is based on patient age, performance status, major comorbidities, psychosocial status, and patient preference (with consideration for disease risk). JAK inhibitors can be used to reduce splenomegaly and MF symptoms as a bridge to transplant, and this is most often my preferred approach.

For patients who are ineligible or uninterested in HCT, outcomes are often better when active treatment is started as soon as possible (Table 2). COMFORT-I showed that patients receiving ruxolitinib had improvements in spleen volume and reported improvements in MF-related symptoms, whereas most of those receiving placebo (and therefore delaying active treatment) had worsening spleen volume and symptoms over the same time period [25]. Similarly, nearly all patients treated with ruxolitinib in COMFORT-II had spleen volume decreases, versus only about half of those treated with best available therapy (thereby delaying targeted treatment with a JAK inhibitor) [22]. Earlier treatment with ruxolitinib also provides long-term improvements in OS. A preplanned OS analysis at 51 weeks in the primary COMFORT-I analysis showed a significant survival advantage for patients randomized to receive ruxolitinib versus patients in whom active treatment was delayed by being randomized to receive placebo [25]. Furthermore, even 5 years later, OS remained significantly longer for patients originally randomized to ruxolitinib compared with those who originally received placebo, despite the crossover from placebo to ruxolitinib within 15 months of study initiation [46], indicating that introducing ruxolitinib later could not fully compensate for the initial delay in treatment. Consistent with the COMFORT-I data, a 5-year pooled analysis of COMFORT-I and COMFORT-II showed that OS was longer for patients originally randomized to receive ruxolitinib compared with those who crossed over later from control treatment [26]. In addition to comparisons between ruxolitinib and placebo, direct comparisons between early and late treatment among patients treated with ruxolitinib reinforce the benefits of early treatment. A pooled post hoc analysis of COMFORT reported improved clinical outcomes for those who started ruxolitinib treatment ≤ 12 versus > 12 months from diagnosis, including fewer cytopenias, better spleen responses, and longer OS [24••]. In addition, among those treated with ruxolitinib in COMFORT-I, greater reductions in *JAK2*V617F allele burden were observed in patients who had a shorter versus a longer time from diagnosis [21]. These results from the COMFORT trials are also supported by real-world data. A retrospective chart review study reported that a > 2-year delay in initiating ruxolitinib was associated with a lower probability of spleen response and numerically lower symptom response [23]. These results were reinforced by data from a separate study also showing that ruxolitinib initiation > 2 years from diagnosis was associated with a lower chance of spleen response [27]. The survival benefits of ruxolitinib compared with no treatment or treatment with other agents has also been demonstrated in multiple studies, further underlining the importance of treatment with ruxolitinib in patients with MF [47–49].

Dose optimization is an important consideration in my practice, with a general goal to administer ruxolitinib at doses ≥ 10 mg bid to maximize potential clinical benefit. The observational, longitudinal RUXOREL-MF study showed the impact of ruxolitinib dose on clinical benefit: a shorter OS was associated with doses < 20 mg bid, and a suboptimal starting dose was often associated with patients not reaching the recommended 20-mg bid dose in the study [50]. Analyses from COMFORT-I also underscored that doses ≥ 10 mg bid led to better clinical outcomes, including reductions in spleen volume and symptom improvement [51, 52], and spleen responses to ruxolitinib have separately been demonstrated to correlate with survival [53–55]. An important exception to initiating patients at ruxolitinib doses ≥ 10 mg bid comes from the REALISE study, which showed that patients with anemia (baseline hemoglobin < 10 g/dL) can be effectively treated with a strategy of initiating ruxolitinib treatment at lower doses (10 mg bid) and titrating up based on tolerance after 12 weeks [44, 56]. Additionally, results from the EXPAND study showed that patients with low platelet counts (50–99 × 10^9^/L) could be safely and effectively treated with a higher ruxolitinib dose (10 mg bid) than that in the US product label/package insert [57].

Another reason we favor starting active treatment as early as possible in patients with MF is that it can better position them to remain at an optimal dose for longer periods of time. It is easier to maintain patients on a stable ruxolitinib dose if treatment is initiated before development of severe anemia and thrombocytopenia, which are more typically seen in patients with higher-risk MF and are often managed with dose reductions [58]. Ruxolitinib discontinuation, often due to cytopenias, can lead to a vicious cycle of reinitiating treatment at a lower dose with reduced efficacy [18•, 42, 50, 59]. This helps underline the importance of earlier initiation of ruxolitinib, which may allow for dose maximization and the potential for better molecular responses.

We also advocate for the use of anemia-directed agents such as erythropoietin and analogs (in patients with endogenous erythropoietin < 125 units/L), danazol, or luspatercept in combination with ruxolitinib to try to maintain ruxolitinib dose intensity rather than reduce the dose of ruxolitinib. In the phase 2 study of luspatercept in patients with MF and anemia, most patients were able to maintain their ruxolitinib dose, and some were able to increase it [60, 61]. Zilurgisertib is an investigational inhibitor of activin receptor type 1 (ACVR1) that appears to improve anemia through reduction of hepcidin production by the liver and may become an important partner for ruxolitinib [62].

## Future Directions

The treatment landscape in MF has evolved dramatically in the last 12 years, with 4 JAK inhibitors now approved as monotherapy. Novel combination therapies based on JAK inhibitor backbones may have the potential to provide further opportunity for disease modification, especially if used early in the disease course. The phase 2 MANIFEST study of pelabresib plus ruxolitinib in JAK inhibitor–naive patients reported that this combination was well tolerated and provided durable improvements in spleen volume and MF symptoms. Of note, the study population had a shorter duration from diagnosis to treatment than historical controls (i.e., many patients received early treatment), which may have enhanced the clinical benefit [63]. Approximately one-quarter of enrolled patients had int-1 MF, and spleen and symptom response rates were similar between patients with int-1 versus int-2 or higher. Based on these results, the phase 3 MANIFEST-2 trial comparing pelabresib plus ruxolitinib versus ruxolitinib monotherapy has been initiated [64]. The first results from this large, placebo-controlled trial (*N* = 430 randomized) were presented at the 65th annual meeting of the American Society of Hematology [65]. Of note, 59% of patients had DIPSS intermediate-1 risk disease. Ruxolitinib plus pelabresib led to a 66% SVR35 rate at 24 weeks, compared with only 35% in the ruxolitinib plus placebo group. A 50% reduction in MPN-SAF total symptom score (TSS50) was achieved by 52% of patients in the combination therapy group and 46% of patients in the ruxolitinib plus placebo group (no statistically significant difference). Additionally, 40% of patients receiving the combination versus only 19% receiving ruxolitinib plus placebo achieved both SVR35 and TSS50 at Week 24. Patients with intermediate risk disease, comprising 94% of the enrolled population, had a statistically significant reduction in absolute mean MPN-SAF total symptom score. Anemia benefits of the combination were also seen. Overall, the combination of ruxolitinib plus pelabresib was well tolerated, with less than 10% of patients discontinuing ruxolitinib due to adverse events. The results of TRANSFORM-1, a phase 3, placebo-controlled trial of navitoclax (*N* = 252), demonstrated a doubling of the SVR35 rate at 24 weeks (63% for ruxolitinib plus navitoclax versus 32% for ruxolitinib plus placebo), although the combination failed to improve symptoms over ruxolitinib alone [66]. However, this trial predominantly accrued patients with DIPSS-plus intermediate-2 and high-risk disease. Outcomes from these and other rational combination therapy trials that aim to achieve “disease modification” beyond that achievable with ruxolitinib alone may further underscore the importance of intervening with effective therapy earlier in the disease course.

## Conclusions

In my practice, we prefer to provide active treatment for patients with MF as early as possible. By initiating treatment earlier in the disease course (i.e., int-1–risk patients), particularly with ruxolitinib in those exhibiting symptoms, patients often experience higher efficacy and lower toxicity compared with starting treatment at a more advanced stage of MF. Combination treatment with ruxolitinib and pegylated interferon alfa-2a has also been investigated in patients with low/int-1–risk MF and was shown to be efficacious and well tolerated in some patients, although this regimen is still best applied in the clinical trial setting. The clinical benefit of initiating treatment with ruxolitinib in patients with int-2 or high-risk MF as soon as possible after diagnosis, rather than delaying treatment, has been demonstrated in the COMFORT studies as well as in other clinical trials and real-world studies. Furthermore, ruxolitinib can be administered long term, with the opportunity to reintroduce ruxolitinib or use different JAK inhibitors sequentially if discontinuation is required, emphasizing that active treatment options are not “used up” by starting early. Taken together, there is little to no benefit to delaying active treatment in patients with symptomatic lower-risk or int-2/high-risk MF.

## Data Availability

No datasets were generated or analyzed during the current study.
